# Cross-linking mass spectrometry for structure analysis of the intrinsically disordered Tau and phosphorylated Tau protein

**DOI:** 10.1371/journal.pcbi.1013868

**Published:** 2026-01-14

**Authors:** Cristian Arsene, Alexander Gates, Anne-Katrin Römmert, André Märtens, Valentina Faustinelli, Luise Luckau, Gavin O’Connor

**Affiliations:** 1 Biochemistry Department, Physikalisch-Technische Bundesanstalt (PTB), Braunschweig und Berlin, Germany; 2 School of Data Science, University of Virginia, Charlottesville, Virginia, United States of America; 3 National Measurement Laboratory, LGC, Guildford, United Kingdom; Indonesia International Institute for Life Sciences, INDONESIA

## Abstract

We present a novel method for analyzing the folding of intrinsically disordered proteins (IDPs), such as Tau and phosphorylated Tau (pTau), in solution. Using cross-linking mass spectrometry (XL-MS) combined with a new downstream analysis framework, we construct weighted interaction networks from cross-link–derived residue pairs without relying on predefined secondary structure assumptions. Structural differences between protein conformations are quantified by comparing the organization of loop structures within their cross-link networks. Validation with bovine serum albumin (BSA) in native and denatured states shows that at least 500 cross-links—requiring 5–10 replicate measurements—are needed for reliable detection of structural divergence. Leave-one-out analysis confirms that structural transitions are global, highlighting the importance of comprehensive cross-link datasets. The coverage of unique cross-links was evaluated using accumulation curves from randomized permutations. Saturation levels were found to be 9.7%, 5.0%, and 6.2% of the total 528 and 10,731 possible cross-links after 30, 84, and 62 technical replicates, respectively, for myoglobin, native BSA, and denatured BSA. For Tau and pTau, coverage reached 10.8% and 5.5% of the upper limit (8,256). Finally, applying our structural analysis to Tau and pTau during arachidonic acid–induced aggregation revealed distinct patterns of structural evolution between the two proteins.

## Author summary

Intrinsically disordered proteins (IDPs) play key roles in cellular regulation and disease, yet their structural characterization remains challenging due to their dynamic and non-canonical conformations. In this study, we present a novel approach for analyzing the folding behavior of IDPs in solution, using Tau and phosphorylated Tau (pTau) as model systems. By refining an existing cross-linking mass spectrometry (XL-MS) protocol and developing a downstream analysis pipeline, we constructed weighted interaction networks from cross-link–derived residue pairs without relying on predefined secondary structure assumptions. These networks are transformed into loop-based structural maps that represent conformational states, enabling their unsupervised clustering and pairwise comparison. Validation with native and denatured bovine serum albumin (BSA) demonstrates the method’s sensitivity to global structural transitions. We further evaluated cross-link coverage saturation using randomized accumulation curves for myoglobin, BSA, and denatured BSA. Applying this framework to Tau and pTau during arachidonic acid–induced aggregation reveals distinct patterns of structural evolution. The method allows for moderate-resolution structural insights into IDPs, which are otherwise inaccessible by traditional techniques such as X-ray crystallography or cryo-EM.

## Introduction

Structural characterization is particularly challenging for intrinsically disordered proteins, whose conformations often cannot be resolved by conventional techniques such as X-ray crystallography or cryo-EM. In these cases, alternative approaches are required to probe three-dimensional organization, even if only at moderate spatial resolution. Cross-linking mass spectrometry (XL-MS) has emerged as one such powerful method, enabling the analysis of protein structures directly in solution by capturing residue–residue proximities across heterogeneous conformational ensembles [1]. Intrinsically disordered proteins tend to aggregate under pathological conditions and are involved in the development of neurodegenerative diseases. Neurofibrillary tangles of the aggregated Tau protein in brain cells are a hallmark of Alzheimer disease and other tauopathies [2]. In this context, hyperphosphorylation of Tau is discussed as a trigger for misfolding of the protein and the formation of oligomers, protofibrills and finally insoluble neurofibrillary tangles. For biomarker proteins of neurodegenerative diseases, including Alzheimer‘s disease, it is important to understand the reason for changes in three-dimensional structure and their impact on the tendency of such proteins to aggregate and to form cytotoxic intracellular neurofibrillary tangles.

Here, we propose a XL-MS based approach for the identification of discrepancies in the higher-order structure of proteins. To illustrate this method, we utilise an example of the phosphorylation status of a recombinant Tau protein in solution, distinguishing between phosphorylated (pTau) and non-phosphorylated states (Tau). To implement this approach, an existing XL-MS protocol was refined to maximize cross-link coverage while minimizing the number of replicate measurements [3]. This enabled the detection of moderate yet otherwise inaccessible structural information, such as distance constraints and conformational features of proteins in solution. XL-MS provides an ensemble-averaged view of protein conformations by capturing diverse cross-linking patterns across many individual molecules. BSA was selected as a model protein for XL-MS method development due to its well-characterized three-dimensional structure, making it ideal for benchmarking cross-linking workflows [4]. Horse myoglobin, with its limited theoretical number of cross-linkable sites, was additionally employed to evaluate the saturation behavior of cross-link detection across replicate experiments. To validate the structural analysis concept, BSA was subjected to controlled conditions designed to induce conformational changes. A covalently binding cross-linker was used to “freeze” the induced structures and bottom-up proteomics was adapted for the analysis of cross-linked peptides. To capture the topological organization of cross-link constraints in BSA, we represented cross-linked residue positions as intra-molecular structural networks for both the native and denatured states. In these networks, nodes correspond to residues and edges encode either sequential adjacency along the primary amino-acid sequence or cross-link–derived long-range interactions, thereby integrating local chain connectivity with cross-link–imposed structural contacts. We then identified all network loops—corresponding to structural motifs of the primary structural sequence constrained by cross-link proximity—and compared their organization between the two states. Loops play a critical role in defining protein fold topology and flexibility, serving as hinges that connect secondary structures and often mediate conformational transitions or ligand binding [5,6]. The set of amino acid residue memberships within a loop is akin to a partition of the network into overlapping clusters, where loop endpoints are shared between adjacent segments. Pairwise comparison of loop organization reveals both global changes in network topology and localized rearrangements along the sequence, highlighting segments that undergo conformational transitions. Tracking loop-membership differences between states pinpoints residues whose long-range contacts are rewired, providing sensitive markers of switching, misfolding, or epitope exposure. We applied this measurement principle to phosphorylated and non-phosphorylated Tau in solution: changing folding states were probed by analyzing aliquots at defined time points during in-vitro aggregation. Loop clusters from the initial cross-link network served as the reference and were compared pairwise to loop clusters derived from subsequent time points, quantifying the emergence, disappearance, splitting, and merging of structural clusters over the aggregation trajectory.

## Methods

**Cross-link detection saturation analysis across replicated measurements.** To assess cross-linking site coverage across measurements, replicates of datasets were randomly permuted and processed to extract non-homeotypic cross-links. After filtering, site-pairs were converted to unique combinations and counted cumulatively over increasing numbers of technical replicates. This was repeated across 84 cycles (for native BSA), and the mean number of detected unique cross-links was computed. Parallel simulations using randomized site-pair permutations were performed over 199 cycles (for native BSA) to estimate stochastic accumulation of cross-links under uniform random sampling. Both empirical and simulated distributions were visualized using Matplotlib with incremental sampling plotted as red and black scatterlines, respectively.

**Parsing of cross-links.** From the list of identified non-homeotypic cross-linked peptides the pairwise cross-linked lysine, serine, threonine and tyrosine positions within the protein sequence were parsed and merged into a list of cross-linked sequence sites. Lists for BSA and Tau were utilized to align with the crystal structure of BSA or with structural models of Tau, and subsequently used for visualization in PyMOL.

**Visualization of protein structures.** The crystal structure of BSA and the Tau structure models with the alignment of cross-links was visualized using PyMOL 1.8 [7]. Structure data files for BSA and the Tau-DMD model were pdb_00004f5s and pdb_00008zzx, respectively. The AlphaLink2 model of Tau and the alignment of cross-links was realized using AlphaLink2 [8].

**Loop-based clustering similarity of protein structures.** Using experimentally determined crosslink-derived residue pairs and the inherent connectivity of amino acid sequences, weighted interaction networks were constructed in Python using the NetworkX package [9]. In these representations, each node corresponds to an amino acid residue, while edges capture two complementary types of interactions: 1) Sequential adjacency, connecting residues that are covalently linked in the primary sequence, representing the peptide backbone; and 2) Cross-link interactions, connecting residue pairs identified experimentally via XL-MS, representing long-range spatial contacts. Edge weights were optionally assigned according to the size (length) of the DSBU crosslinker enabling distance constraints of 26-30 Å between C-*α* atoms or the Euclidean distance inferred from structural models when available. The resulting graphs thus integrate both the linear topology of the peptide chain and experimentally constrained tertiary contacts.

Loop structures were then defined as cyclic subgraphs formed when cross-link edges connect non-adjacent residues along the primary sequence, enclosing one or more intervening sequence segments. Each loop represents a constrained structural motif—analogous to a local folding unit or hinge—bounded by the cross-linked residues. All unique loops were extracted by tracing the shortest path along the primary-sequence backbone between cross-linked nodes. This operation yields a set of overlapping loops, where residues at loop termini can participate in multiple loops, reflecting shared structural participation in different folding motifs. The collection of loop memberships therefore constitutes a soft (overlapping) clustering of residues across the protein sequence.

To quantify structural differences between protein states (e.g., native vs. denatured BSA, or Tau vs. pTau), loop-based clusterings were compared using an element-centric similarity (ECS) measure (*α* = 0.9) implemented in the CluSim Python package [10,11]. Unlike global metrics such as RMSD that compare atomic coordinates, ECS evaluates the consistency of residue co-membership—that is, whether two residues belong to the same loop (i.e., share a constrained structural environment) across conditions. Each residue’s similarity score reflects its local reorganization: residues that maintain loop relationships between conditions have high similarity, while those that shift between loops or lose connectivity exhibit low similarity. ECS also offers two practical advantages: (i) it naturally scales with loop size, so changes confined to small loops contribute proportionally less to the global score than reorganizations of large loops; and (ii) it provides attribution of disagreement, enabling us to localize differences to specific residues or cohesive residue groups (i.e., particular loops or loop subsections). The tuning parameter *α* controls the relative emphasis on local versus global agreement; *α* = 0.9 favors detection of subtle, localized changes typical of partial unfolding or phosphorylation-induced rearrangements. This framework allows structural changes to be quantified directly from cross-link–derived contact information without requiring atomic-level models.

This approach assesses the agreement in residue co-membership across conditions, penalizing or rewarding reassignments depending on their local neighborhood context. For robustness assessment, bootstrap resampling (n = 10) was performed across increasing subsets of cross-link data to evaluate the stability of the derived clustering structure. Mean similarity scores and associated 95% confidence intervals were then computed to characterize both intra- and inter-condition variability.

The resulting similarity metrics were visualized as error-bounded line plots, showing the evolution of structural agreement across samples and conditions. These plots highlight the degree of global topological change as well as localized sequence regions undergoing loop reorganization. Together, these analyses provide a reproducible framework for detecting structural rearrangements and conformational transitions from XL-MS data.

**3D Visualization of node-specific similarity in protein networks.** Normalized element-centric similarity scores between loop-clustered native and denatured BSA, as well as Tau and pTau networks, were visualized in three-dimensional space. Weighted multigraphs derived from cross-linking data (stored in GML format) were processed using NetworkX. Node positions were determined by a spring-layout embedding (dim = 3, iterations = 1000), with edge lengths modulated to reflect cross-link distance constraints. To ensure reproducible node placement, a fixed random seed (seed = 27061971) was applied to the layout algorithm. The 3D embedding is an abstract, topology-preserving layout rather than an atomistic model; it does not encode Cartesian coordinates. However, because spring forces are weighted by cross-link constraints and sequential connectivity, the layout preserves relative proximity of loop neighborhoods and global contact topology. Consequently, clusters and spatial adjacencies in the visualization correspond to regions with dense cross-linking and shared loop memberships, providing an interpretable map of contact reorganization across conditions while acknowledging the absence of atomic resolution.

Residue-specific similarity scores (*α* = 0.9) computed with CluSim were mapped to node colors using a plasma colormap and visualized interactively with Plotly. Short- and long-range edges were rendered separately to emphasize structural organization, and every tenth node was annotated to assist spatial orientation and interpretation.

**Estimation of the compactness of the BSA loop-network.** The loop-network was modeled as a weighted graph using NetworkX, where backbone connections were fixed at 3.8 Å and cross-links were randomly assigned distances within defined ranges. For each cross-link distance range, 20 independent simulations were performed, with cross-link lengths randomly permuted using a uniform distribution to reflect structural variability. A 3D spring layout algorithm was used to generate spatial configurations, and node positions were scaled to preserve average backbone spacing. The radius of gyration (Rg) was estimated for each simulated structure to quantify spatial compactness. The Rg value of the BSA crystal structure was calculated from the Cartesian coordinates of C-*α* atoms by measuring their spatial dispersion around their geometric center.

**Leave-one-out analysis of cross-link influence.** To evaluate the structural impact of individual cross-links unique to native BSA (Tau), a leave-one-out approach was applied. Each cross-link was removed from the native BSA (Tau) network one at a time, and the resulting network was reanalyzed to identify changes in loop topology. The modified network was compared to both the original native BSA (Tau) and denatured BSA (pTau) networks using element-wise clustering similarity scores (CluSim, *α* = 0.9).

**Data availibility.** The mass spectrometry proteomics data and the scripts have been deposited to the ProteomeXchange Consortium via the PRIDE partner repository with the dataset identifier PXD067659.

## Results and discussion

### Identification of cross-links and distance-constraints.

**Fig 1:**
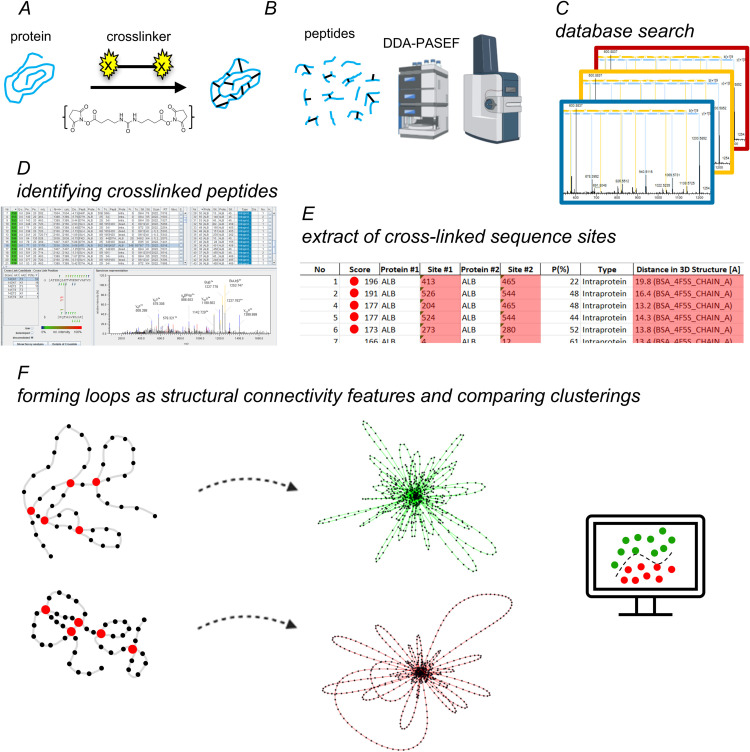
Workflow for cross-linking MS and data analysis. A: The pure protein is cross-linked by reaction with DBSU. B: The cross-linked protein is digested and tryptic peptides are subjected to data dependent analysis using high-resolution mass spectrometry (icon created in https://BioRender.com). C: Cross-linked peptides are analysed by data base search. D: Cross-linked peptides are identified based on default statistical criteria. E: Cross-linked sequence sites are filtered from the result list of data analysis for exclusion of homeotypic interactions and decoy entries. If available, data from X-ray crystallography can be used to compare cross-linked sequence sites, allowing distance constraints to be extracted from the results list. F: Construction of structural cross-link networks and pairwise comparison of the loop-based clusterings.

The analytical workflow commenced with a cross-linking reaction followed by trypsin digestion of the modified protein. Building upon an established XL-MS protocol [3], liquid chromatographic conditions were further refined to enhance the reliable detection of cross-linked peptides across a larger number of technical replicates. Cross-linked peptides were identified using MEROX 2.0, which performs database-assisted search for precursor- and fragment ions [12]. Pairwise cross-linked sequence sites were extracted from the resulting dataset. The connectivity between adjacent amino acids along the protein backbone and the experimentally derived cross-links between lysines, serines, threonines and tyrosines was used to build the cross-link structural network and extract loop-based clusterings. The workflow as illustrated in Fig 1, was validated using horse myoglobin and BSA.

**Fig 2:**
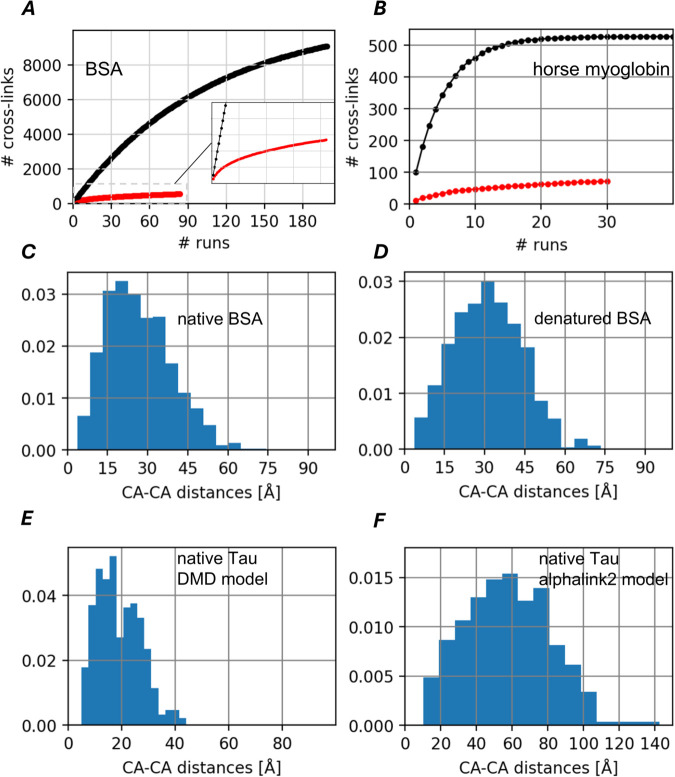
Accumulation of unique cross-links across technical replicates and the distribution of distance constraints. A and B: The number of identified unique cross-links (red line) was accumulated across randomized permutations of technical replicates of the same cross-linked and digested protein. The black line shows the expected accumulation of unique cross-links based on a random sampling model, assuming 100 randomly selected links per cycle from a fixed pool of possible cross-links. C-F: Distributions of distance constraints were derived by comparing the identified cross-linked sequence sites from native and denatured BSA and from native Tau to their corresponding Euclidean distances between respective C-alpha atoms in the X-ray crystal structure of BSA or in the Tau conformer, either predicted by discrete molecular dinamics (DMD) simulation or by AlphaLink2.

The maximum number of cross-links between lysines, serines, threonines and tyrosines is 528 and 10731 in myoglobin and albumin, respectively. However, only a small fraction of 9.7% and 5.0% of the total number of cross-links was identified across 30 and 84 technical replicates in denatured myoglobin and in native BSA, respectively (Fig 2A, 2B). To overcome the limited detection of cross-linked peptides in single MS runs, observed in our optimized protocol for myoglobin and BSA and consistent with Belsom et al. [13], each sample was analyzed in at least 30 technical replicates. This approach balanced analysis time with data completeness, allowing software-assisted identification of the majority of cross-linked amino acid site pairs within the protein sequence (Fig 2A, 2B).

**Fig 3:**
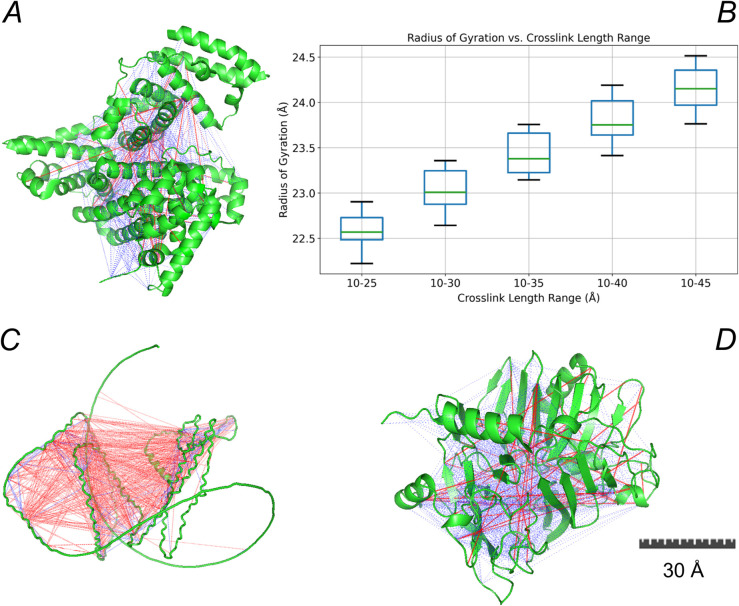
Protein folding structures with identified cross-links and compactness of the BSA structural network. A: x-ray crystal structure of BSA. Blue and red lines show the cross-links. B: Simulated radius of gyration (Rg) of BSA structural networks across varying cross-link length ranges. Each box represents 20 independent simulations using a 3D spring layout model with weighted crosslinks. C and D: Alphalink2 and DMD structure model of Tau, respectively. Lines highlighted in blue indicate distances < 40 Å between the C-*α* atoms of pairwise cross-linked amino acids. Red lines indicate distances > 40 Å.

Duplicates were not eliminated, since the amount of duplicates contains chemical information about accessibility of reaction sites for cross-linking and therefore structural information. As a test for the analysis of structural changes of the protein folding, BSA was analysed in its native form and after protein denaturation. In denatured BSA, as with native BSA, only 6.2% of the total cross-links were detected across 62 technical replicates (S1 Fig). The identified cross-links in both native and denatured BSA were aligned with the distances between cross-linked sequence sites derived from the X-ray crystal structure of native BSA [14]. The distributions of these distance constraints are shown in Fig 2C, 2D. Compared to native BSA, the distribution for denatured BSA is shifted toward longer distances between cross-linked sites. This indicates a more flexible and extended conformation of the denatured protein. Considering the molecular size of the crosslinker and the limited flexibility of native BSA due to its 17 disulfide bridges, the identified cross-links closely matched the spatial distances between the C-*α* atoms of the corresponding amino acid pairs in the X-ray crystal structure (Fig 3A). The red lines representing distances greater than 40 Å suggest a degree of structural flexibility in the protein at room temperature in solution, as they exceed the maximum span of the DSBU cross-linker, which is approximately 30 Å [15].

Attemps were made to obtain higher-order structural insights in protein folding by integrating XL-MS data with structural predictions resulting in the development of AlphaLink2 [16]. However, the analysis of the intrinsically disordered Tau protein using AlphaLink2 predominantly yields, as already expected from alphafold prediction [17–19], a random coil conformation, with only a few short helical segments (Fig 3C). Discrete molecular dynamics (DMD) simulations of the Tau conformer was also combined with cross-linking data resulting in a predicted globular protein model as shown in Fig 3D [20]. In comparison to the AlphaLink2 model which we obtained by alignment of our own cross-linking data with the predicted protein conformer, the globular Tau model displays only limited regions of unstructured folding. Its predominantly globular structure and the abundance of secondary structural elements do not adequately account for the intrinsic flexibility of Tau, which is critical for its dynamic association and dissociation with neuronal microtubules under physiological conditions. The resulting distribution of distance constraints for the Alphalink2 structure model was broad, consistent with the behavior expected from a highly flexible protein conformation. For comparison we alligned our cross-linking data also with the globular Tau model predicted by DMD simulation, resulting in a narrow distribution of short distance constraints (Fig 2E, 2F).

In terms of conformational dynamics, the DMD model more closely resembles the compact, globular structure of BSA than the extended and flexible Alphalink2 configuration of Tau, which, notably, aligns well with cross-links that exceed the maximum span of the DSBU cross-linker (Fig 3C). A resolution to this inconclusive scenario may be achieved by obviating the need for assumptions regarding secondary structure, which are often difficult—or even impossible—to make for intrinsically disordered proteins.

### Topological similarity of protein structures.

We now recast the structural comparison of intrinsically disordered proteins in terms of the topoligical similarity of loop clustering motifs induced by cross-links within a topological network model. Rather than assuming atomic coordinates, cross-links between non-adjacent residues delineate constrained sequence segments (loops) whose organization reflects higher-order structure. Using a standard 3.8 Å spacing between adjacent amino acids, the compactness of the cross-link structural network, constructed for native BSA was compared to its crystal structure. The cross-link structural network was simulated as a weighted graph with fixed backbone links and randomly assigned cross-link distances. For each range, 20 simulations were run to reflect structural variability. Spatial models were generated and the radius of gyration was calculated to assess compactness. Cross-link alignment with the crystal structure resulted in an average distance of 21.7 ± 10.9 Å with 45 Å marking the 95th percentile. Considering the natural flexibility of the protein, which can shift some distance constraints beyond 30 Å, a Rg value of 24.2 Å was considered appropriate (Fig 3B). The Rg of the crystal structure was 26.75 Å. Overall, both the cross-link structural network and the crystal structure exhibit comparable levels of compactness.

**Fig 4:**
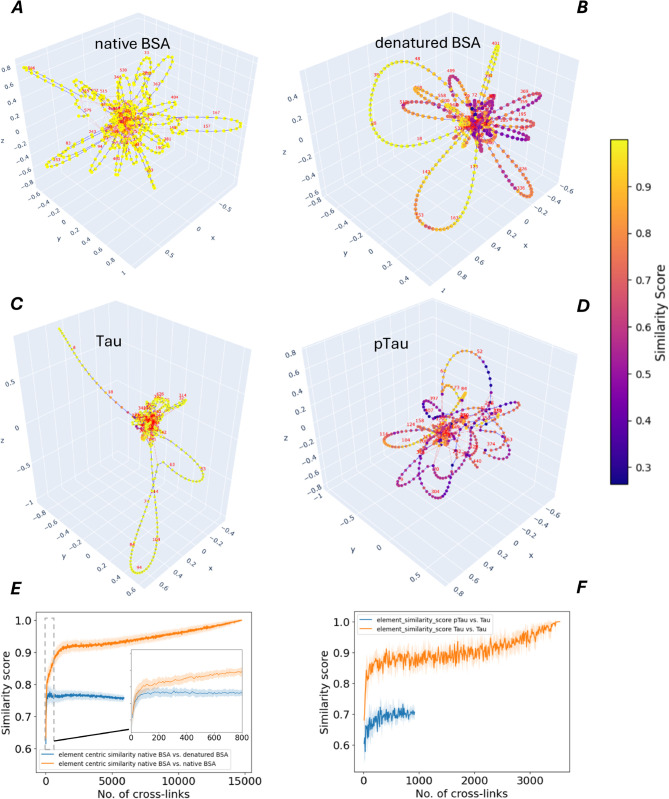
Cross-link networks of BSA, Tau, and pTau conformers, and the element-centric similarity of their loop clusterings. A–D: Clustering similarity based network visualizations of cross-link networks for BSA, Tau, and pTau conformers, with pairwise comparisons of native vs. denatured BSA using native BSA as the reference (A,B) and Tau vs. pTau using Tau as the reference (C,D). Structural differences are highlighted via element-wise similarity scores between loop clusterings. Red edges denote cross-links (edge length= 8), and blue edges represent sequential (primary sequence) connections (edge length = 1). E and F: Element-centric similarity of loop clusterings derived from cross-link networks of native vs. denatured BSA (E) and Tau vs. pTau (F). Similarity scores are shown as a function of sample size, averaged over randomized subsamples of cross-links; shaded areas represent standard deviation.

We next compared the loop-based clusterings from the BSA cross-link structural networks in the native and denatured states (Fig 4A, 4B). The resulting residue-level similarity map shows a global reduction in concordance between states, consistent with the disruption of long-range constraints and extensive reorganization of tertiary contacts (Fig 4E). The residue-wise profiles highlight localized hotspots of change concentrated at loop termini and hinge-like segments, indicating splitting, merging, or disappearance of native loops. Correspondingly, large native loops tend to fragment into multiple shorter loops or dissolve, while some previously unconstrained segments gain new, short-range loops characteristic of expanded, partially unfolded conformations. Together, these patterns indicate that denaturation involves selective rewiring of loop memberships rather than a uniform loss of structural constraints, thereby offering a mechanistic description of higher-order structural degradation.

Similarily, cross-link data obtained for Tau and pTau were used to construct the respective topological networks and extract their loop clustering structure, which were analyzed in the same manner as the BSA networks (Fig 4C, 4D).

### Sample size and associated error for pair-wise comparison of clusterings.

It is important to note that the number of cross-links retained when constructing the structural networks strongly influences the pairwise comparison of loop-network clusterings. To determine the lower bound on the number of cross-links required to detect divergence between conformers (native vs denatured BSA) and between Tau and pTau, we generated randomized cross-link subsamples of increasing size, built the corresponding structural networks, and then clustered their loop-networks for pairwise comparison (Fig 4E, 4F). For BSA, we estimate that a minimum of 500 cross-links (including duplicate observations) is required to reliably detect structural divergence between the two conformers. Given a typical yield of 50–100 cross-links per technical replicate, this corresponds to approximately 5–10 replicates to achieve sufficient coverage.

### Impact of individual cross-links on structural differences

**Fig 5:**
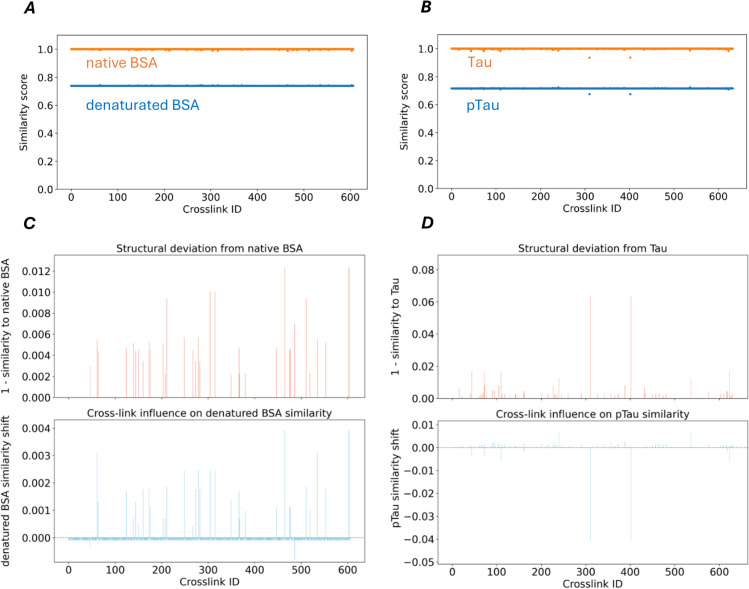
Leave-one-out analysis of native BSA- or Tau-specific cross-links and their impact on loop-based clustering similarity to denatured BSA or pTau. A and B: Distributions of similarity scores across sequence positions using a leave-one-out model, where for BSA each native-specific cross-link and for Tau each Tau-specific cross-link is removed to assess its influence on clustering similarity to both protein pairs (native and denatured BSA, or Tau and pTau). C and D: Differential impact of native BSA- or Tau-specific cross-links on loop-based clustering similarity to native and denatured BSA or Tau and pTau. Bar plots illustrate the structural influence of BSA- and Tau-specific cross-links based on loop-based clustering similarity metrics. Top panels: Deviation from the original BSA or Tau loop-based clustering upon individual cross-link removal, expressed as 1 - similarity. Bottom panels: Leave-one-out similarity shift relative to denatured BSA or pTau, indicating whether removal of a cross-link increases or decreases alignment with the denatured BSA or pTau loop-based clustering. This differential analysis builds on the raw similarity scores shown in A and B.

To assess whether specific cross-links drive the main differences between native and denatured BSA, we focused on the structural cross-link network of native BSA and first identified cross-links that are absent in the denatured state. These native-specific cross-links were then removed one at a time in a leave-one-out scheme, and for each perturbed cross-link network we recomputed the corresponding loop-based clustering. The resulting loop-based clusterings were compared both to the original native clustering and to that of denatured BSA, yielding residue-wise similarity profiles along the sequence (Fig 5A).

Using the leave-one-out approach developed for the BSA networks, we evaluated the influence of individual cross-links on structural similarity between Tau and pTau. Notably, removal of two Tau-only cross-links reduced the similarity of the Tau loop network to its original form and, unexpectedly, also decreased its similarity to the pTau loop network. Given that these cross-links were considered key differentiators between the two networks, we had anticipated that their removal would increase similarity to pTau, not decrease it. Plots of element-similarity scores for both BSA and Tau (Fig 5A, 5B) indicate that such changes affect the protein’s overall structure, underscoring the need for a holistic analysis of cross-links to accurately capture structural features. The limited subset of cross-links detectable in a single measurement is insufficient for this purpose. In Tau and pTau, only 10.8% and 5.5% of the maximum possible cross-links (8,256) were detected across 100 and 120 technical replicates, respectively (S1 Fig). The removal of an individual cross-link from the loop-network of native BSA or Tau results in an average loop-based clustering similarity shift of only 0.03% and 0.06% per link, respectively, when compared to the original loop-network. When the modified loop-network is instead compared to that of denatured BSA or pTau, the average similarity shift is 0.02% and 0.05% per link. The differential impact of single native BSA- or Tau-specific cross-links on loop-network similarity to native and denatured BSA or Tau and pTau is shown in Fig 5C, 5D.

### Structural changes of Tau and pTau in the run-up to aggregation.

**Fig 6:**
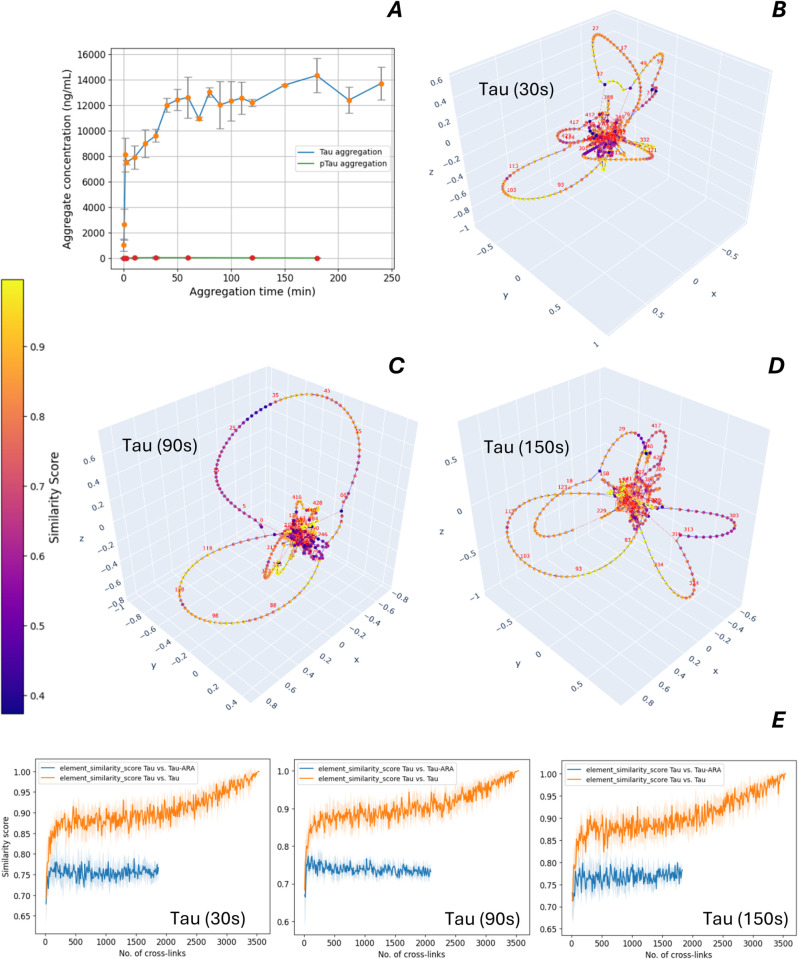
Aggregation and structure analysis of Tau conformers. A: Time course of Tau and pTau aggregation, induced by arachidonic acid. An ELISA was used to quantify the tau aggregates. Mean values are shown with error bars corresponding to the standard deviation (2 runs). B–D: Cross-link network visualizations for Tau at different time points during aggregation. E: Similarity scores for the loop clusterings as a function of sample size, comparing the cross-link network of each Tau conformer (aggregated for different durations) to the reference Tau network obtained prior to aggregation with arachidonic acid. Scores are averaged over randomized subsamples of cross-links; shaded areas represent standard deviation.

The aggregation of Tau and pTau was induced by addition of arachidonic acid (ARA) as previously described [21]. For monitoring the time course of aggregation, a Tau aggregate ELISA was used, which sensitively detects oligomers and aggregates [22] (S1 File). The epitope of the assay antibody is located in the Tau amino acid sequence region of 428 to 437. While the aggregation of Tau is saturated after 2 hours reaction time, the aggregation of pTau is inhibited (Fig 6A). There is no direct interference of the assay antibody with the phosphorylation sites, which may explain the very low ELISA results for pTau aggregates, since our pTau material is uniformly threetimes phosphorylated at T169, S199 and T217, while the epitope of the assay-antibody is far away from this region. At the beginning of the aggregation, aliquots were taken and immediately cross-linked for structural analysis. The networks and element-centric similarities of tau cross-linked at different time points during aggregation, and compared pairwise with tau cross-linked before the start of aggregation, are shown in Fig 6B–6E. Tau showed early structural alterations during aggregation that continued to be detectable over time. The cross-linking reaction was optimized for the formation of intra-molecular cross-links by chosing a low protein concentration. However, during aggregation, the formation of intermolecular cross-links within oligomers of Tau cannot be omitted. Therefore, aliquots for cross-linking were taken only at the beginning of the aggregation within 2-3 min of the reaction time when oligomers and aggregates are not yet formed.

**Fig 7:**
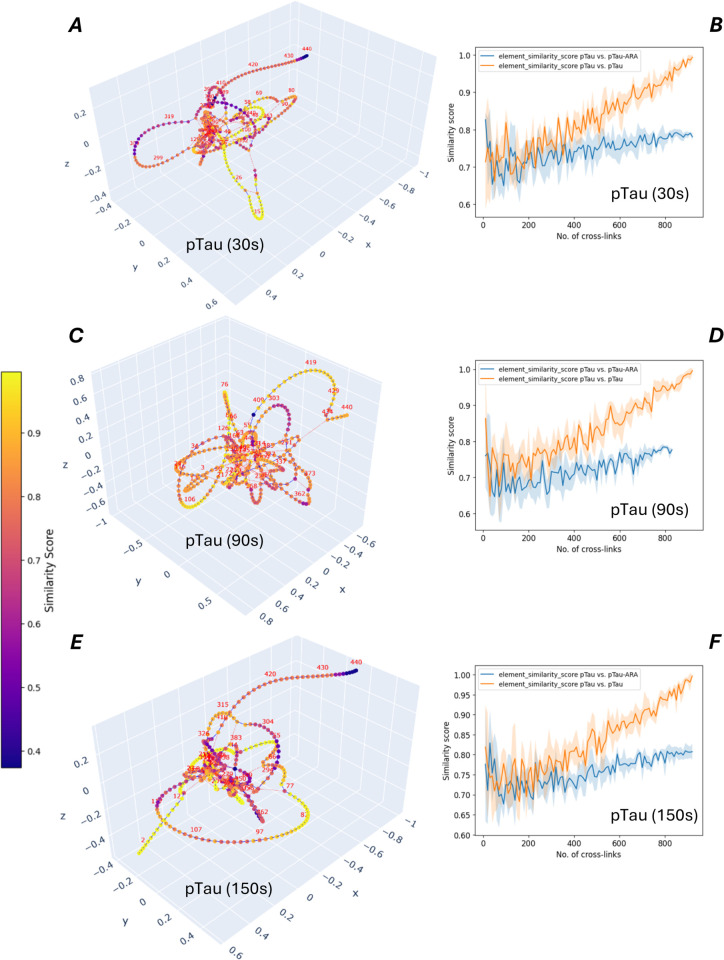
Aggregation and structure analysis of pTau conformers. A, C and E: Cross-link structural network visualizations of cross-linked pTau at different time points of aggregation. B, D and F: Loop-clustering similarity scores are shown across sample sizes for the comparison of the network of each pTau conformer, cross-linked at different time points of aggregation, and the reference network for pTau, cross-linked at the beginning before starting the aggregation with arachidonic acid. Similarity scores were averaged over randomized subsamples of cross-links. Shaded areas indicate standard deviation.

The same analysis method was applied to pTau and its aggregation kinetics. If compared to the aggregation of Tau, only small changes of the structure were detected during the preliminary phase before aggregation (Fig 7A–7F). In agreement with XL-MS, circular dichroism spectroscopy in the pre-aggregation phase detected structural changes in Tau, but not in pTau (S2 Fig). In contrast to our triphosphorylated pTau material, the hyperphosphorylated Tau, which was aggregated under similar conditions (75μM ARA, 2μM Tau or pTau) in a previuos study [23], showed very similar aggregation kinetics as unphosphorylated Tau. However, the electrostatic environment of at least 12 phosphorylated sites across the seqence of the hyperphosphorylated Tau influences the higher-order protein structure and its flexibility most probably in a different way, resulting even in the opposite aggregation behavior, if compared to our triphosporylated Tau material.

## Conclusion

The new cross-linking analysis method enables the sensitive detection and relative quantification of structural divergence between protein conformers under different conditions and over time. This opens the possibility to further investigate the influence of post-translational modification (PTM) patterns e.g. the differential phosphorylation of Tau on the relationship between structure and aggregation.

## Supporting information

S1 Fig Accumulation of unique cross-links across technical replicates. The number of identified unique cross-links (red line) was accumulated across randomized permutations of technical replicates of the same cross-linked and digested protein. The black line shows the expected accumulation of unique cross-links based on a random sampling model, assuming 100 randomly selected links per cycle from a fixed pool of possible cross-links.
(TIFF)

S2 Fig Far-UV circular dichroism (CD) spectra of Tau and pTau measured over time during incubation with arachidonic acid (ARA). CD spectra of Tau reveal rapid structural transitions within minutes of ARA-induced aggregation, characterized by a shift of the spectral minimum to 213 nm, consistent with *β*-sheet formation. In contrast, phosphorylated Tau (pTau) spectra remain unchanged under identical conditions, indicating the absence of comparable structural rearrangements.
(TIFF)

S1 File Materials and chemical analysis methods. (PDF)
