# Supplementary figures and images for: Cross-linking mass spectrometry for structure analysis of the intrinsically disordered Tau and phosphorylated Tau protein
Source: PLoS Comput Biol. 2026 Jan 14;22(1):e1013868. doi: 10.1371/journal.pcbi.1013868 (PMC12826526; doi:10.1371/journal.pcbi.1013868)

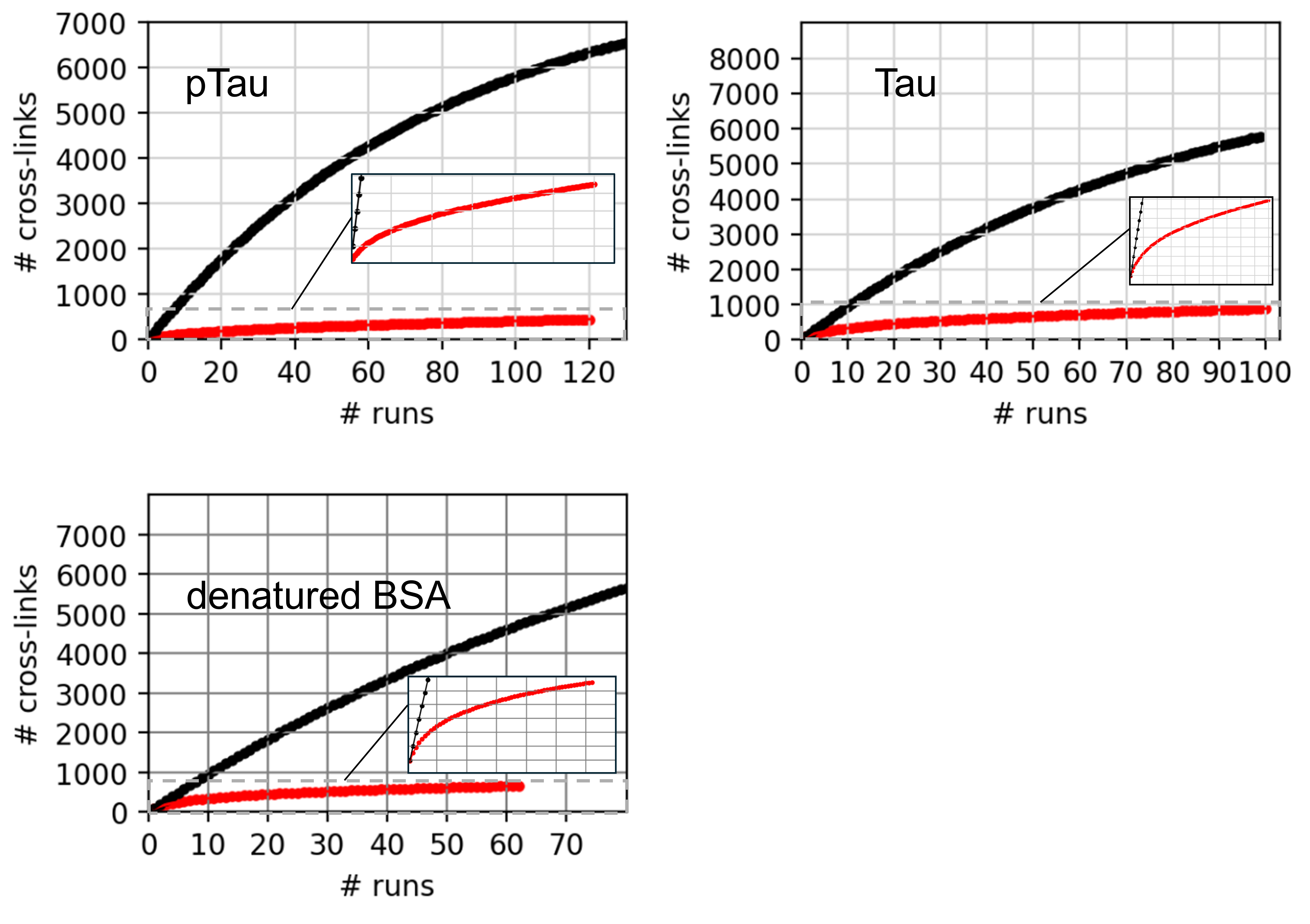

Supplement: S1 Fig — The number of identified unique cross-links (red line) was accumulated across randomized permutations of technical replicates of the same cross-linked and digested protein. The black line shows the expected accumulation of unique cross-links based on a random sampling model, assuming 100 randomly selected links per cycle from a fixed pool of possible cross-links. (TIFF) [file pcbi.1013868.s001.tif]

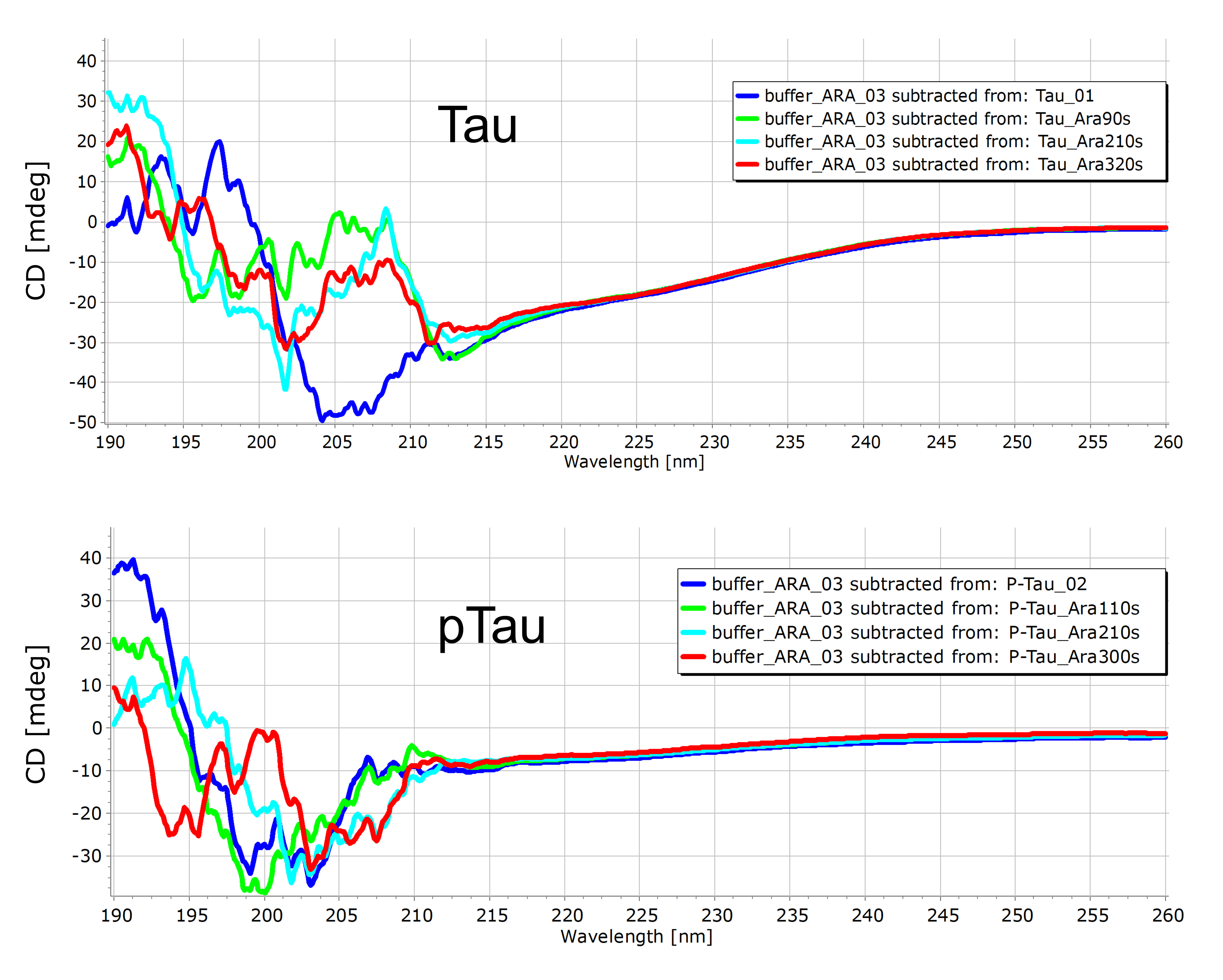

Supplement: S2 Fig — CD spectra of Tau reveal rapid structural transitions within minutes of ARA-induced aggregation, characterized by a shift of the spectral minimum to 213 nm, consistent with β-sheet formation. In contrast, phosphorylated Tau (pTau) spectra remain unchanged under identical conditions, indicating the absence of comparable structural rearrangements. (TIFF) [file pcbi.1013868.s002.tif]
