# Supplementary material for: Cross-linking mass spectrometry for structure analysis of the intrinsically disordered Tau and phosphorylated Tau protein
Source: PLoS Comput Biol. 2026 Jan 14;22(1):e1013868. doi: 10.1371/journal.pcbi.1013868 (PMC12826526; doi:10.1371/journal.pcbi.1013868)
Supplement: S1 File — (PDF) [file pcbi.1013868.s003.pdf]

Supporting Information: Cross-linking mass spectrometry for structure analysis of the intrinsically disordered Tau and phosphorylated Tau protein

Cristian Arsene,[†] Alexander Gates,[‡] Anne-Katrin Römmert,[†] André Märtens,[†] Valentina Faustinelli,[¶] Luise Luckau,[¶] and Gavin O'Connor[*,†]

†*Biochemistry Department, Physikalisch-Technische Bundesanstalt (PTB), Braunschweig und Berlin, Germany*

‡*School of Data Science, University of Virginia, Charlottesville, Virginia, United States of America*

¶*National Measurement Laboratory, LGC, Guildford, United Kingdom.*

E-mail: christian.arsene@ptb.de

Contents

Protein materials S-3

Aggregation of Tau and pTau. S-4

Circular dichroism spectroscopy during aggregation of Tau and pTau. . . S-4

Cross-linking. S-5

Proteolysis. S-5

Liquid chromatography-mass spectrometry. S-6

Database search for precursor and fragment ions. S-6

Protein materials

Native BSA. BSA was purchased from Sigma-Aldrich, St. Louis, USA, cat. no.: 05470. A solution of 10 μM BSA in 1 mL of 50 mM 4-(2-Hydroxyethyl)-1-piperazine ethanesulfonic acid (HEPES)-buffer (pH7.5) was prepared and filtered by centrifugation at 7000 x g through a 2 mL-Amicon Ultra centrifugal filter (10 kDa MWCO). The residue on the Amicon filter was washed 5 times with 50 mM HEPES-buffer, and finally dissolved in 1 mL of 50 mM HEPES-buffer and transferred to cross-linking.

Denatured BSA. For the structure analysis of denatured BSA, an additional solution of 10 μM BSA was prepared in HEPES-buffer. For reduction of disulfide bridges, an aliquot of 5 μL of an aqueous 34 mM tris(2-carboxyethyl)phosphine (TCEP) solution was added and the protein solution was incubated for 1 hour at 60 ℃. For alkylation, an aliquot of 10 μL of an aqueous 34 mM 2-iodoacetamide (IAA) solution was added and the protein solution was incubated for 30 min at room temperature. This solution was filtered through a 2 mL-Amicon Ultra centrifugal filter and washed as the native-BSA solution.

Myoglobin. Myoglobin from horse skeletal muscle was obtained from SIGMA-Aldrich, St. Louis, USA, cat. no.: 70025). A solution in 50 mM HEPES-buffer was prepared as described for denatured BSA. Myoglobin was only used in denatured form for method validation, in order to maximize the number of accessible reaction sites for the cross-linking reaction.

Tau and phosphorylated Tau (pTau). Recombinant Tau and pTau (GSK-3β-phosphorylated) were from Acro Biosystems, Basel, Switzerland. Both materials were obtained as solutions of 0.5 mg/g in 50 mM Tris, 150 mM NaCl, 1 mM DTT, 1 mM EDTA, pH 7.5. The phosphorylation pattern of pTau was analysed using a standard bottom-up proteomics protocol, as recently reported.[S1] Phosphorylation was used as variable modification for database search. Intact protein mass spectrometry was used to investigate the distribution of the number of phosphorylations in pTau, as previously described.[S2] The material contained unphosphorylated Tau beside uniformly triphosphorylated Tau. Intact protein mass spectrometry revealed a deconvoluted mass of 45718.7 and 45953.4 Da, for Tau and pTau respectively. Ex-

pected masses were: 45716.7 (Tau) and 45958.7 Da (pTau). The pTau material was further purified using strong-anion exchange chromatography on a MONO-Q 4.6/100 PE column. Gradient elution was applied within 42 min between 0 and 20% NaCl in a 20 mM Tris-buffer. The flow rate was 1mL/min. Fractions containing pTau were collected. The stock solutions of Tau and the purified pTau were filtered and washed in the same way as the BSA solution. Aliquots of Tau or pTau solution containing $\sim$ 1 nmol of the protein were submitted to aggregation reaction and cross-linking.

Aggregation of Tau and pTau

For aggregation, 2 mL of a solution of 2 μM Tau or pTau were prepared in a buffer, containing 50 mM HEPES and 100 mM NaCl at pH 7.5. A sample (500 μL) was taken and the aggregation was started by addition of 2 mM arachidonic acid (ARA) in ethanol to the remaining Tau or pTau solution to adjust a target ARA concentration of 75 μM. Additional samples (500 μL) were collected at 30, 90 and 150 sec following the initiation of the aggregation. From each sample, an aliquot of 10 μL was taken for the Tau aggregate ELISA.[S3] Each remaining sample was diluted in 500 μL of the 50 mM HEPES buffer, containing 100 mM NaCl and submitted to the cross-linking reaction.

Circular dichroism spectroscopy during aggregation of Tau and pTau.

Tau and pTau solutions were examined in buffer containing 50 mM HEPES and 100 mM NaCl (pH 7.5), at protein concentrations of 0.5 and 0.3 mg/mL, respectively. Aggregation was triggered through the addition of ARA (2 mM in ethanol), resulting in a final concentration of 75 μM. The measurements were carried out at different time points of Tau or pTau aggregation in a quartz cuvette (1 mm) in Jasco J-810 CD spectro-polarimeter (Jasco, Tokyo, Japan). The scan was carried out at a speed of 200 nm/min in the range of 190-260 nm at 1 nm band-width. Protein spectra were background-corrected using spectra of buffer solution containing 75 μM ARA.

Cross-linking

The cross-linking reagent disuccinimidyl dibutyric urea (DSBU) was obtained from ThermoFisher Scientific, Darmstadt, Germany, cat. no.: A35459. A stock solution of 25 mM DSBU in 94 μL of dried DMSO was prepared. To each filtered protein solution or aliquot from aggregation solution a 20 μL-aliquot of the cross-linker stock solution was added and gently shaked for 1 hour at room temperature. The reaction was stopped by addition of 20 μL of 1M ammonium hydrogen carbonate solution. The solution was confined under vacuum at room temperature and submitted to proteolysis.

Proteolysis

To the product of the cross-linking reaction 25 μL of denaturation buffer were added, containing 8M urea in 400 mM ammonium hydrogen carbonate. The solution was denatured for 5 min in an ultrasonic bath. For reduction of disulfide bridges, an aliquot of 8 μL of an aqueous 20 mM DTT-solution was added and the reaction solution was incubated for 30 min at 60 ℃. For alkylation, an aliquot of 10 μL of an aqueous 60 mM IAA-solution was added and the reaction solution was incubated for 20 min at room temperature. The excess of IAA was quentched by addition of 36 μL of a 20 mM DTT-solution. Pure water (160 μL) was added to lower the urea concentration to 1 M and the proteolysis was started by addition of 13 μL of a solution containing trypsin at 1μg/μL. An additional aliquot of 13 μL of the trypsin solution was added after 24 hours of incubation at 37 ℃ and the proteolysis was continued over night. The product was desalted on solid phase cartridges following a standard protocol using two 5 mM ammonium acetate buffers: an aqueous buffer for the wash step and a solution of 80% acetonitrile for the elution of peptides. The eluate was confined under vacuum and the residue of 60 μL was submitted to LC-MS/MS analysis.

Liquid chromatography-mass spectrometry

An UltiMate 3000 RSLCnano HPLC system (ThermoFisher Scientific) coupled to a timsTOF Pro mass spectrometer (Bruker Daltonics) was used for the analysis of the proteolysed sample of cross-linked protein. Aliquots of 2 μL were analysed. Peptides were trapped on a pre-column (Acclaim PepMap C18, 5 μm, 0.3x5 mm) and then separated on a Bruker Fifteen nanoFlow column (25 cm x 150 μm, C18, 1.9 μm, 120 Å) using a water-acetonitrile gradient from 2 to 17% B in 55 min, from 17% to 25% B in 30 min then from 25% to 37% B in 10 min and from 37% to 90% B in 10 min (with solvent A: water, 0.1 vol.-% formic acid and B: acetonitrile, 0.1 vol.-% formic acid) at 50 °C. The flow rate was 700 nl/min. The timsTOF Pro mass spectrometer was equipped with a CaptiveSpray ion source. The mass spectrometer was run using the DDA-PASEF-standard-1.1 sec-cycletime method, as provided by Bruker with some modifications. Briefly, the settings were: 14 PASEF MS/MS scans per acquisition cycle with a trapped ion mobility accumulation and elution time of 166 ms. The charge state minimum and maximum for precursor ions was set to 3 and 8, respectively. Spectra were acquired in a m/z range of 100 to 1700 and in an (inverse) ion mobility range ($1/K_0$) of 0.60 to 1.60 Vs/cm^2. The collision energy was set up as a linear function of ion mobility starting from 20 eV for $1/K_0$ of 0.6 to 95 eV for $1/K_0$ of 1.6.

Database search for precursor and fragment ions

MeroX 2.0,[S4] Version 2.0.1.4 was used for identification of cross-linked peptides. Databases for *BSA*, *horse myoglobin* and *Tau* were obtained as FASTA files (uniprot.org, P02769, P68082 and P10636-8, respectively for BSA, horse myoglobin and microtubule-associated human protein tau, accessed: 18. Apr. 2023). The following settings were applied for data analysis: carbamidomethylation of cysteine as fixed modification, methionine oxidation and phosphorylation of amino acids serine, threonine or tyrosine as variable modifications. Proteolytic cleavage sites were R and K and DSBU was set as cross-linking reagent for the reaction at side chains of amino acids lysine, serine, threonine and tyrosine and at the N-terminus of

the protein. The mass tolerance for the monoisotopic mass of precursor ions and fragment ions was 15 and 25 ppm, respectively. Only $b-$ and $y-$ fragment ions were considered. Mass correction was enabled for precursor ions. The minimum score for cross-linked peptides was set to 20. The false discovery rate (FDR) was 1%. Lists of identified cross-linked peptides including the cross-linked amino acid positions within the protein sequence were used for downstream data analysis.

References

(S1) Henrion, A.; Arsene, C.-G.; Liebl, M.; O'Connor, G. Label-free quantification of host cell protein impurity in recombinant hemoglobin materials. *Analytical and Bioanalytical Chemistry* **2024**, *416*, 387–396.

(S2) Coppens, S.; Gogishvili, D.; Faustinelli, V.; Scollo, E.; Hopley, C.; Abeln, S.; Dalby, P.; Goenaga-Infante, H.; Luckau, L.; Vialaret, J.; Lehmann, S.; Hirtz, C.; Illes-Toth, E. Neurofilament Light Chain under the Lens of Structural Mass Spectrometry. *ACS Chemical Neuroscience* **2025**, *16*, 141–151.

(S3) Roboscreen Human TAU AGGREGATE ELISA: Enzyme immunoassay for quantitative determination of human TAU aggregates. `https://www.roboscreen.com/products/neurodegeneration/human-tau-aggregate-elisa/`, 2021.

(S4) Götze, M.; Pettelkau, J.; Fritzsche, R.; Ihling, C. H.; Schäfer, M.; Sinz, A. Automated assignment of MS/MS cleavable cross-links in protein 3D-structure analysis. *Journal of the American Society for Mass Spectrometry* **2015**, *26*, 83–97.
